# Echolocating Whales and Bats Express the Motor Protein Prestin in the Inner Ear: A Potential Marker for Hearing Loss

**DOI:** 10.3389/fvets.2020.00429

**Published:** 2020-07-17

**Authors:** Maria Morell, A. Wayne Vogl, Lonneke L. IJsseldijk, Marina Piscitelli-Doshkov, Ling Tong, Sonja Ostertag, Marisa Ferreira, Natalia Fraija-Fernandez, Kathleen M. Colegrove, Jean-Luc Puel, Stephen A. Raverty, Robert E. Shadwick

**Affiliations:** ^1^Zoology Department, The University of British Columbia, Vancouver, BC, Canada; ^2^Inserm Unit 1051, Institute for Neurosciences of Montpellier, Montpellier, France; ^3^Institute for Terrestrial and Aquatic Wildlife Research, University of Veterinary Medicine Hannover, Foundation, Büsum, Germany; ^4^Department of Cellular and Physiological Sciences, Life Sciences Institute, The University of British Columbia, Vancouver, BC, Canada; ^5^Division of Pathology, Department of Biomolecular Health Sciences, Faculty of Veterinary Medicine, Utrecht University, Utrecht, Netherlands; ^6^Virginia Merrill Bloedel Hearing Research Center, University of Washington, Seattle, WA, United States; ^7^Department of Fisheries and Oceans Canada, Winnipeg, MB, Canada; ^8^Marine Animal Tissue Bank, Portuguese Wildlife Society, Estação de Campo de Quiaios, Figueira da Foz, Portugal; ^9^Centro Reabilitação Animais Marinhos, CPRAM, Ecomare, Estrada Do Porto de Pesca Costeira, Gafanha da Nazaré, Portugal; ^10^Marine Zoology Unit, Cavanilles Institute of Biodiversity and Evolutionary Biology, Science Park, University of Valencia, Valencia, Spain; ^11^Zoological Pathology Program, University of Illinois at Urbana-Champaign, Brookfield, IL, United States; ^12^Animal Health Center, Ministry of Agriculture, Abbotsford, BC, Canada

**Keywords:** prestin, hair cells, inner ear, bat, whale, echolocation, noise-induced hearing loss, immunofluorescence

## Abstract

Prestin is an integral membrane motor protein located in outer hair cells of the mammalian cochlea. It is responsible for electromotility and required for cochlear amplification. Although prestin works in a cycle-by-cycle mode up to frequencies of at least 79 kHz, it is not known whether or not prestin is required for the extreme high frequencies used by echolocating species. Cetaceans are known to possess a prestin coding gene. However, the expression and distribution pattern of the protein in the cetacean cochlea has not been determined, and the contribution of prestin to echolocation has not yet been resolved. Here we report the expression of the protein prestin in five species of echolocating whales and two species of echolocating bats. Positive labeling in the basolateral membrane of outer hair cells, using three anti-prestin antibodies, was found all along the cochlear spiral in echolocating species. These findings provide morphological evidence that prestin can have a role in cochlear amplification in the basolateral membrane up to 120–180 kHz. In addition, labeling of the cochlea with a combination of anti-prestin, anti-neurofilament, anti-myosin VI and/or phalloidin and DAPI will be useful for detecting potential recent cases of noise-induced hearing loss in stranded cetaceans. This study improves our understanding of the mechanisms involved in sound transduction in echolocating mammals, as well as describing an optimized methodology for detecting cases of hearing loss in stranded marine mammals.

## Introduction

The mammalian cochlea contains two types of auditory sensory cells, the inner hair cells (IHCs) and the outer hair cells (OHCs) that are arranged in one single row of IHCs and three rows of OHCs along the organ of Corti, or hearing organ. While OHCs amplify the incoming sound stimulation within the cochlea and are essential for the exquisite frequency selectivity and sensitivity, IHCs transduce the acoustic stimulation into the release of glutamate onto the afferent auditory nerve fibers. To achieve these tasks, both hair cell types differ in their molecular and protein composition.

Prestin is the motor protein of OHCs that is responsible for electromotility (changes in length) and required for cochlear amplification (1). Prestin is a member of the membrane transporter superfamily of SLC26A proteins. It is expressed on the whole basolateral membrane of OHCs of terrestrial mammals (2, 3) and undergoes a conformational change at a high rate when detecting changes in the membrane potential [(4), reviewed in (5–7)].

In response to incoming sound stimulation, the stereociliary bundle on the apical surfaces of the OHCs is deflected, leading to the opening of the mechano-electrical transduction channels. The cation influx through these channels then depolarizes the cells, which results in a conformational change of prestin. Because of the high density of prestin within the basolateral membrane [estimated to be 8,400 motor elements/μm^2^, (8)], depolarizing and hyperpolarizing current injection, shortens and lengthens the OHCs, respectively. Thus, the electromotile response of OHCs enhances the membrane basilar motion in response to sound stimulation. The targeted deletion of prestin in mice results in loss of OHC electromotility and a 40–60 dB loss of hearing sensitivity by the cochlea (9), as well as a loss of frequency selectivity (10).

Although prestin works in a cycle-by-cycle mode up to frequencies of at least 79 kHz (11, 12), it is not known whether prestin is required for the extreme high frequencies used by echolocating species.

Echolocating whales are known to possess a prestin coding gene that shows a sequence convergence with the prestin gene in echolocating bats (13–15). However, the expression and distribution patterns of the protein in the cetacean and bat cochleas have not been determined, and the contribution of prestin to echolocation has not yet been resolved.

Toothed whales, or odontocetes, are cetaceans that echolocate and, depending on the species, are able to produce and hear acoustic signals typically up to 120–180 kHz. Audiograms measured on these species indicate different low threshold hearing ranges, including 125 Hz for beluga whales [*Delphinapterus leucas*, (16)], 150 Hz for bottlenose dolphins [*Tursiops truncatus*, (17)], and 250 Hz both for striped dolphins [*Stenella coeruleoalba*, (18)] and for harbor porpoises [*Phocoena phocoena*, (19)]. The upper end of the hearing range of echolocating bats is similar to the lower ranges of toothed whales, extending from 5.2 kHz up to 150 kHz for Seba's short-tailed bat [*Carollia perspicillata*, (20)] and up to 111 kHz for Parnell's mustached bat [*Pteronotus parnellii*, (21)]. Currently, we do not know if echolocating species express prestin or if the protein is required to transduce the very high frequencies.

There is an increasing concern about how noise pollution might affect hearing in cetaceans. Exposure to high intensity sound for long periods of time can damage the auditory neurons and the hair cells of the organ of Corti and can ultimately lead to the hair cell death by apoptosis. Since cochlear hair cells do not regenerate in mammals, the neighboring supporting cells actively participate in the process of hair cell elimination and “scar” formation. The scarring comprises the simultaneous expansion and sealing of the reticular lamina (22–24) as a rapid protective response to hair cell apoptosis. The presence of scarring among hair cell rows is therefore an important criterion that can be used to assess any possible history of noise-induced hearing loss.

In other mammals that have been studied, such as guinea pigs and mice, the distribution patterns of prestin change as the result of damage to the OHCs. According to Abrashkin et al. (25), clumps of prestin are found up to at least 9 days after noise or ototoxic drug exposure in the cytoplasm of supporting cells. Previous studies on guinea pigs, mice, humans, chinchillas, monkeys and cats also showed swelling of the afferent nerve endings on the IHCs with incipient retrograde nerve degeneration (26) and loss of spiral ganglion cell bodies and primary auditory neurons after acoustic overstimulation (27–29).

The current optimized protocol to visualize scars in the cochlea of stranded cetaceans using scanning electron microscopy (SEM) allows a very high definition of the cuticular plate and the possibility to distinguish between hair cell death from post-mortem decomposition artifacts (30, 31). However, it is not possible to visualize the sensory cell body and auditory innervation with this particular dissection for SEM. In addition, it is extremely challenging to determine the age of a lesion when found with SEM. Consequently, there is a need to describe a method optimized for cetacean cochlea that allows the visualization of the hair cells and supporting cells of the organ of Corti, as well as type I afferent innervation, that permits distinguishing between newly formed and old lesions. Thus, if prestin is expressed in the OHCs of cetaceans, we could use its labeling as a marker for recent cases of noise-induced hearing loss. In addition, it is well-described how scars are formed and how to distinguish their shape over the first 9 h of exposure in the guinea pig using phalloidin labeling (32). In cases of recent hair cell death, anti-prestin antibody and phalloidin labeling would help explaining potential causes of death of stranded cetaceans. In some cases, there are no apparent gross or microscopic lesions, which may have accounted for the loss of the stranded animal and diagnostic testing do not identify any specific pathogen or toxin. In these cases, the analysis of the inner ear would be particularly helpful in better understanding the potential contribution of noise-induced hearing loss to the stranding. Additionally, in all cases, the analysis of inner ears aids in evaluating effects of noise pollution (as well as other etiologies) on cetacean hearing.

In this study, we report the presence of the motor protein prestin all along the cochlear spiral in five species of echolocating whales (harbor porpoise, bottlenose dolphin, common dolphin, striped dolphin and beluga whale) and two species of echolocating bats (Parnell's mustached bat and Seba's short-tailed bat). Our conclusions are based on immunofluorescence staining patters using three different antibodies, two that recognize the n-terminus and one antibody against the c-terminus of the protein prestin. In addition, we present a protocol that can be used to distinguish between newly formed and old lesions combining several antibodies to label the cells of the organ of Corti and associated innervation.

## Materials and Methods

### Sample Collection

Nineteen ears from five odontocete species were perfused perilymphatically between 1 and 16 h post-mortem with 10% neutral buffered formalin or 4% paraformaldehyde following the protocol by Morell and André (33). The variability on the delay in fixation was due to the opportunistic nature of cetacean samples. Specifically, cochleas were collected and preserved from stranded harbor porpoises (*Phocoena phocoena, n* = 12 ears, one individual from a rehabilitation facility), bottlenose dolphin (*Tursiops truncatus, n* = 2), common dolphin (*Delphinus delphis, n* = 1), striped dolphin (*Stenella coeruleoalba, n* = 1), and beluga whales from oceanaria and sustainably harvested (*Delphinapterus leucas, n* = 3). The details on the origin of the samples and cause of death are given in Table 1.

**Table 1:** Details of the origin and species of the inner ear samples processed for this study, as well as the number of hours between the death of each animal and fixation of its cochlea, the cause of death and the time used to decalcify the periotic or *petrossus* bone with ethylenediaminetetraacetic acid.

**Id**	**Species**	**Common name**	**Origin**	**Age group**	**Time death-perfusion**	**Time decal.**	**Cause of death**
Cet 303A_UT993	*Phocoena phocoena*	Harbor porpoise	The Netherlands	Neonate	5–6 h	52 days	Emaciation and acute starvation
Cet 305A_UT1005	*Phocoena phocoena*	Harbor porpoise	The Netherlands	Adult	5–6 h	78 days	Infectious disease
Cet 352B_UT1318	*Phocoena phocoena*	Harbor porpoise	The Netherlands	Neonate	7–8 h	36 days	Acute starvation
Cet 353B_UT1331	*Phocoena phocoena*	Harbor porpoise	The Netherlands	Neonate	5–6 h	37 days	Asphyxiation following bycatch
Cet 399B_UT1485	*Phocoena phocoena*	Harbor porpoise	The Netherlands	Juvenile	10 h	21 days	Asphyxiation following bycatch
Cet 400B_UT1486	*Phocoena phocoena*	Harbor porpoise	The Netherlands	Juvenile	15 h	27 days	Infectious disease
Cet 401B_Pp02	*Phocoena phocoena*	Harbor porpoise	The Netherlands	Adult	16 h	42 days	Not available
Cet 404B_UT1495	*Phocoena phocoena*	Harbor porpoise	The Netherlands	Juvenile	4 h	43 days	Emaciation of unknown origin
Cet 413A_UT1535	*Phocoena phocoena*	Harbor porpoise	The Netherlands	Adult	4 h	43 days	Infectious disease
Cet 426A_UT1562	*Phocoena phocoena*	Harbor porpoise	The Netherlands	Adult	4 h	44 days	Infectious disease
Cet 426B_UT1562	*Phocoena phocoena*	Harbor porpoise	The Netherlands	Adult	4 h	44 days	Infectious disease
Cet 432B_UT1602	*Phocoena phocoena*	Harbor porpoise	The Netherlands	Adult	5.5 h	44 days	Infectious disease
Cet 394A_15-1637	*Tursiops truncatus*	Bottlenose dolphin	United States	Adult	2 h	63 days	Infectious disease
Cet 435A_KLC248	*Tursiops truncatus*	Bottlenose dolphin	United States	Adult	2–3 h	91 days	Infection disease
Cet 314A_DDE_203_2013	*Delphinus delphis*	Common dolphin	Portugal	Juvenile	7 h	92 days	By-catch in beach seine
Cet 385B_SC150819	*Stenella coeruleoalba*	Striped dolphin	Spain	Adult	8 h	32 days	Infectious disease
Cet 340A_HI-14-08	*Delphinapterus leucas*	Beluga whale	Canada	Adult	1.25 h	100 days	Harvested
Cet 419A	*Delphinapterus leucas*	Beluga whale	Canada	Adult	5.5 h	61 days	Infectious disease
Cet 420A	*Delphinapterus leucas*	Beluga whale	Canada	Adult	16 h	57 days	Hypovolemic shock
Bat 03A	*Pteronotus parnellii*	Parnell's mustached bat	Germany	Adult	5 min	29 h	Humanely euthanized
Bat 07A	*Carollia perspicillata*	Seba's short-tailed bat	Germany	Adult	5 min	12.5 h	Humanely euthanized
Bat 08 B	*Carollia perspicillata*	Seba's short-tailed bat	Germany	Adult	5 min	7 h	Humanely euthanized
Bat 11 B	*Carollia perspicillata*	Seba's short-tailed bat	Germany	Adult	5 min	3.5 h	Humanely euthanized

Four cochleas from two species of echolocating bats, Parnell's mustached bat (*Pteronotus parnellii, n* = 1) and Seba's short-tailed bat (*Carollia perspicillata, n* = 3), were perfused intracardially at a rate of 4 ml/min, the first 5 min with 0.9% NaCl, and then for 30 min with 4% paraformaldehyde in 0.1 M phosphate buffer. The samples from bats were provided by Dr. Manfred Kössl (Institute of Cell Biology and Neuroscience, Goethe University, Germany) and euthanized for another study.

All protected cetacean samples were transported with the appropriate CITES permits and all required permits were as follows: Aurora Research Institute License No. 15467, Department of Fisheries and Oceans Fishing License No. S-14/15-3019-YK, Marine Mammal Transport License #18843, and Environmental Impact Screening Committee (EISC) #03-14-03. All experiments with bats were performed in accordance with current laws for animal experimentation in Germany (Regierungspräsidium Darmstadt) and with the declaration of Helsinki.

### Sample Preparation

The periotic, or *petrossus*, bones were decalcified by immersion in 14% ethylenediaminetetraacetic acid (EDTA) tetrasodium salt hydrate (Alfa Aesar or Sigma-Aldrich), pH 7.4, at room temperature [changing the solution once every 7–10 days; (34)]. Table 1 shows the decalcification times. The decalcification time varies depending on the volume of the periotic bone of each species (35).

**Figure 1:**
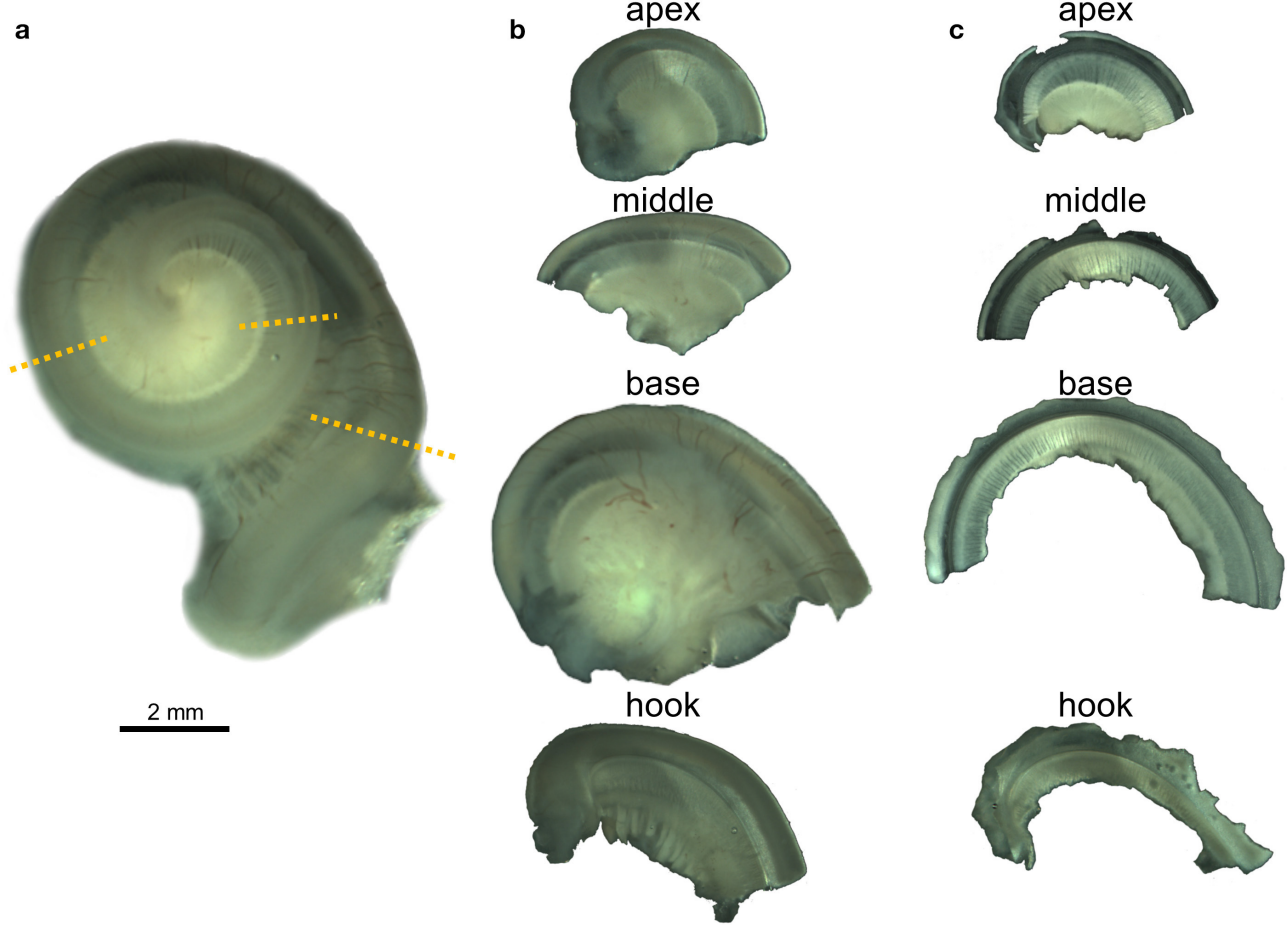
Whole-mount preparation steps illustrated with the right cochlea from the harbor porpoise UT993. **(a)** Cochlea after being decalcified with 14% EDTA. Dotted lines indicate the locations where the cochlea was sectioned. **(b)** Half turns after being sectioned. **(c)** Flat preparations after being dissected.

All 23 cochleas were dissected for immunofluorescence imaging using the whole-mount technique, also called surface preparation, adapting an optimized protocol already described for terrestrial mammals (36). The decalcified periotic bone around the cochlear spiral was removed with microscissors isolating the cochlea. Holding part of the remaining decalcified periotic bone of the base of the cochlea or the modiolus with forceps, the preserved cochleas were transversely sectioned with microscissors into four pieces, starting by the apical turn or apex, then the middle, base and hook subsequently (Figures 1a,b). The four regions were further dissected and the vestibular and tympanic scalas removed until flat preparations of the cochlear epithelium, spiral limbus and Rosenthal canal were obtained (Figure 1c). The Reissner and tectorial membranes were removed, and the spiral ligament trimmed below the *stria vascularis*. The spiral ganglion cells were left in most of the preparations, but the modiolus was removed.

### Immunohistochemical Staining

The cochlear sections were initially blocked with 5% normal donkey serum (Millipore) for 1 h at room temperature, incubated with the primary antibodies overnight at 4°C, washed three times 10 min each with PBT (0.1% triton-X 100 with 2 mg/l bovine serum albumin in PBS) and then incubated with secondary antibodies for 2 h in the dark at room temperature (1:400 dilution), and for 30 min with DAPI (Thermo Scientific ref. 62248, 1:1,000 dilution), which counter-stains the nucleus. The whole-mounts were washed three times 10 min each with PBS. The following primary antibodies were used:

- Goat anti-Prestin polyclonal IgG antibody (Santa Cruz ref. SC-22692). This antibody has been shown by others to be specific for prestin (37, 38), which is present in OHCs basolateral membrane. It recognizes the first 50 amino acids of the n-terminus extreme. A 1:100 to 1:200 dilution was used.
- Rabbit anti-Prestin polyclonal antibody, courtesy of Dr. Jing Zheng's lab (Northwestern University, ref. NW802). This antibody recognizes the n-terminus extreme and has been shown to be specific for the protein prestin (39). A1:1,000 dilution was used.
- Rabbit anti-Prestin polyclonal antibody, courtesy of Dr. Jing Zheng's lab (ref. NW958). This antibody recognizes the c-terminus extreme and has been shown to be specific for the protein prestin (40). A 1: 1,000 dilution was used.
- Rabbit anti-Myosin VI polyclonal antibody (Proteus ref. 25-6791). Myosin VI antibody labels IHCs and OHCs, which we used to identify double labeling of these cell types with prestin antibody. A 1:500 dilution was used.
- Rabbit anti-Myosin VIIa polyclonal antibody (Proteus ref. 25-6790). Myosin VIIa antibody labels IHCs and OHCs. We tested it in one sample in combination with Myosin VI antibody and used a 1:500 dilution.
- Mouse anti-Neurofilament 200 (phosphorylated and non-phosphorylated) monoclonal antibody (Sigma-Aldrich ref. N0142) IgG1 isotope. This antibody labels the intermediate filaments, found in the cytoplasm and axon of neurons and labels type I afferent innervation in the inner ear. Type I afferent innervation comprises 95% of the afferent innervation of the cochlea in some species of odontocetes (41). A 1:400 dilution was used.
- Chicken anti-Neurofilament Heavy polyclonal antibody (Millipore ref. AB5539). This antibody recognizes type I afferent innervation in the inner ear. A 1:5,000 dilution was used.
- Rabbit anti-Neurofilament–L antibody (Cell Signaling Technology ref. C28E10). We used a 1:100 dilution in one sample to determine its specificity for cetacean species.

The secondary antibodies used were Alexa Fluor® 488 donkey anti-goat IgG, Alexa Fluor® 568 donkey anti-rabbit IgG, Alexa Fluor® 647 donkey anti-mouse IgG (Molecular Probes refs. A11055, A10042, A31571, respectively) and Alexa Fluor® 633 donkey anti-chicken IgY (Sigma-Aldrich ref. SAB4600127). DAPI (4′, 6-diamidino-2′-phenylindole, dihydrochloride; Thermo Scientific™ ref. 62247) was used at a 1:1,000 dilution to label nuclei, and phalloidin (FluoProbes® X5 505, ref. FP-AZ0130) was used at 1:100 dilution to label F-actin.

Samples, including the controls, were treated with 0.2% Sudan Black B for 10 min after the secondary antibody to reduce the fluorescence of the tissue. To wash the excess of Sudan Black B, the whole-mounts were rinsed three times of 1 min each with Ethanol 70%, followed by three times of 10 min each with PBS. The flat preparations were individually mounted on a glass slide with 0.1% N-propyl gallate in 90% glycerol and evaluated using fluorescence optic microscope and an Olympus FV1000 confocal microscope at the University of British Columbia Bioimaging Facility (UBC, Vancouver, Canada) and a Zeiss LSM880 confocal microscope at the Montpellier Resources Imagery (MRI, Montpellier Cell Biology Research Center, France).

### Controls

Small subsections from the four large half turns (Figure 1c) were processed as controls. The subsections were taken randomly from regions of the cochlea. Controls included: (1) Control for the specificity of binding by the primary antibody (sections were incubated with normal (non-immune) IgG at the same concentration at which the primary antibody was used—Sigma-Aldrich ref. I5256, M5284, and I5006, and then incubated with the same concentrations of the secondary antibody and DAPI as used on experimental sections); (2) Control for non-specific binding of the secondary antibodies (samples were incubated without the primary antibodies, but with same concentrations of the secondary antibody as used on experimental sections, and DAPI in some cases); (3) Control for autofluorescence (no primary and no secondary antibodies were used).

Micrographs of the three controls were taken using the same settings as their respective treatments (i.e., same magnification and same intensity of the four lasers). Brightness and contrast were enhanced, using identical values for treatments and the respective controls.

## Results

### Echolocating Whales and Bats Express Prestin Along the Entire Cochlear Spiral

**Figure 2:**
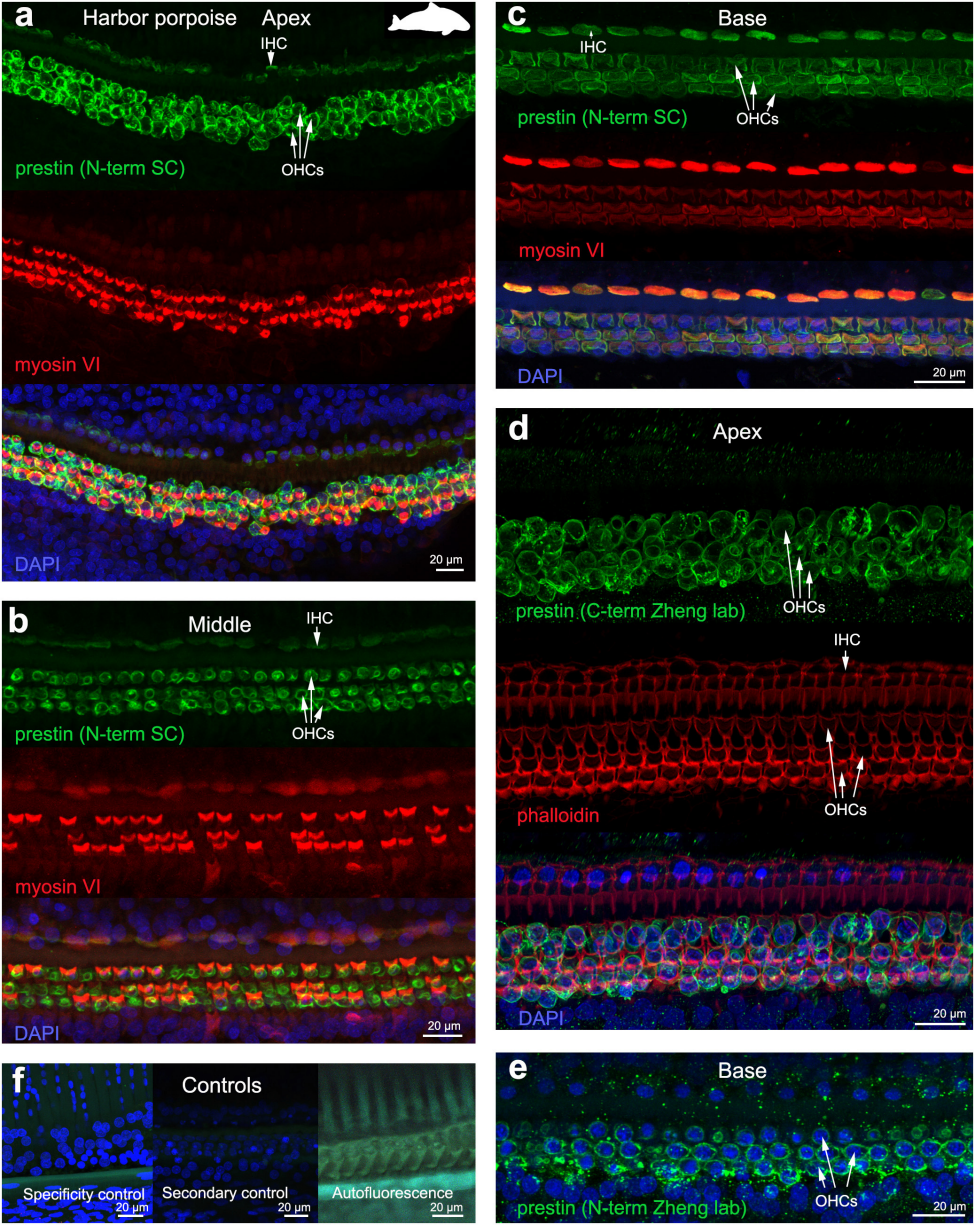
Immunofluorescence images from maximum projections from z stacks from the apex **(a,d)**, middle **(b)** and base **(c,e)** of the cochlea of harbor porpoise *Phocoena phocoena*. We labeled the whole mounts with anti-prestin antibody from Santa Cruz (SC) in **(a–c)** (green), with anti-prestin antibodies from Dr. Zheng lab (green) that recognize the c-terminus **(d)** and n-terminus extreme **(e)** of the protein, anti-myosinVI antibody (red) that labels inner (IHCs) and outer hair cells (OHCs), and DAPI (blue). **(f)** Specificity control, secondary control (control for non-specific binding of the secondary antibodies) and autofluorescence control from **(c)**. The cuticular plate from IHCs and OHCs was positively labeled by anti-prestin antibody from Santa Cruz, but not with anti-prestin antibodies from Dr. Zheng lab. The animal image inserted in **(a)** corresponds to the animal species of cochlea stained by immunofluorescence.

**Figure 3:**
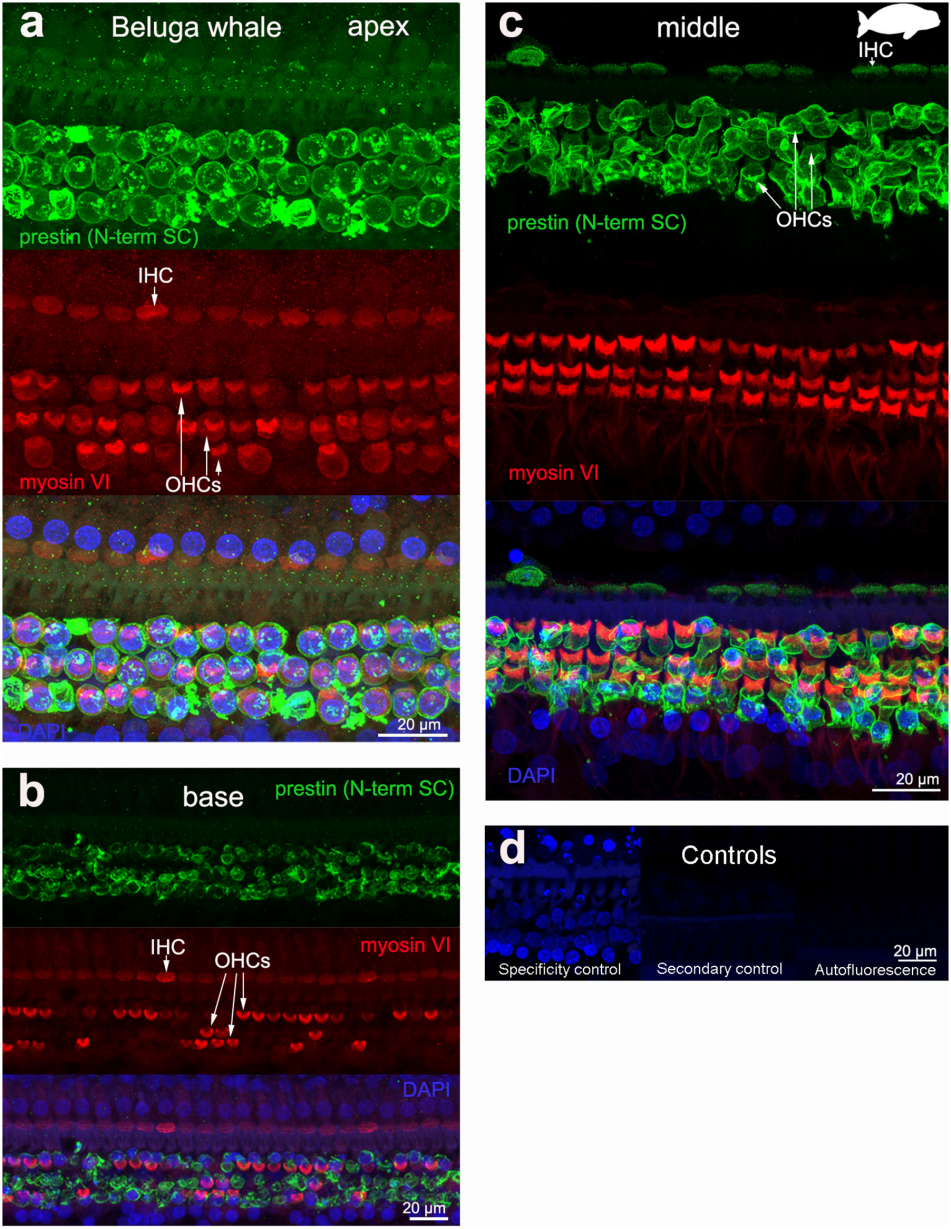
Immunofluorescence images from maximum projections from z stacks from the apical **(a)**, middle **(c)**, and basal **(b)** turn of the cochlea from beluga whale *Delphinapterus leucas*. We labeled the flat preparations with anti-prestin antibody from Santa Cruz (SC, green), anti-myosinVI antibody (red) that labels inner (IHCs) and outer hair cells (OHCs), and DAPI (blue). **(d)** Specificity control, secondary control (control for non-specific binding of the secondary antibodies) and autofluorescence control from image **(c)**. The animal images inserted in **(c)** correspond to the animal species of cochlea stained by immunofluorescence.

**Figure 4:**
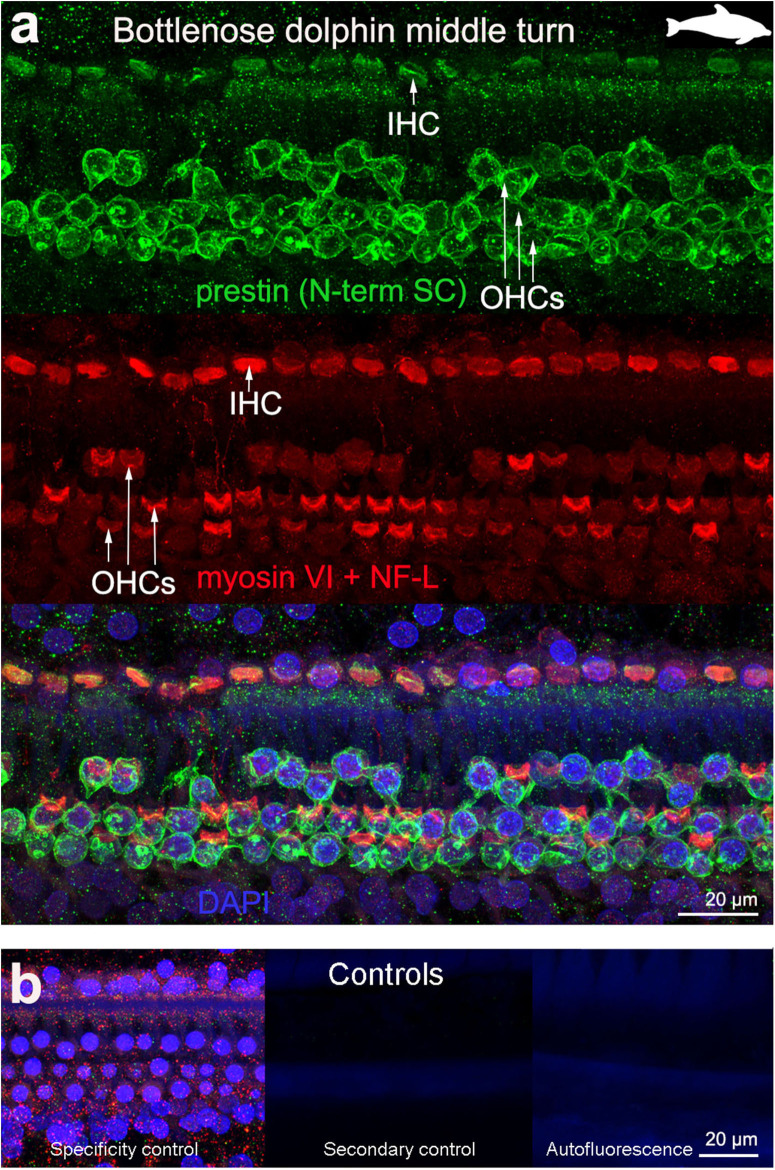
Immunofluorescence images from maximum projections from z stacks from the middle turn of the cochlea from bottlenose dolphin *Tursiops truncatus*. We labeled the flat preparations with anti-prestin antibody from Santa Cruz (SC, green), anti-myosinVI antibody (red) that labels inner (IHCs) and outer hair cells (OHCs), and DAPI (blue). **(b)** Specificity control, secondary control (control for non-specific binding of the secondary antibodies) and autofluorescence control from **(a)**. The animal images inserted in **(a)** correspond to the animal species of cochlea stained by immunofluorescence.

**Figure 5:**
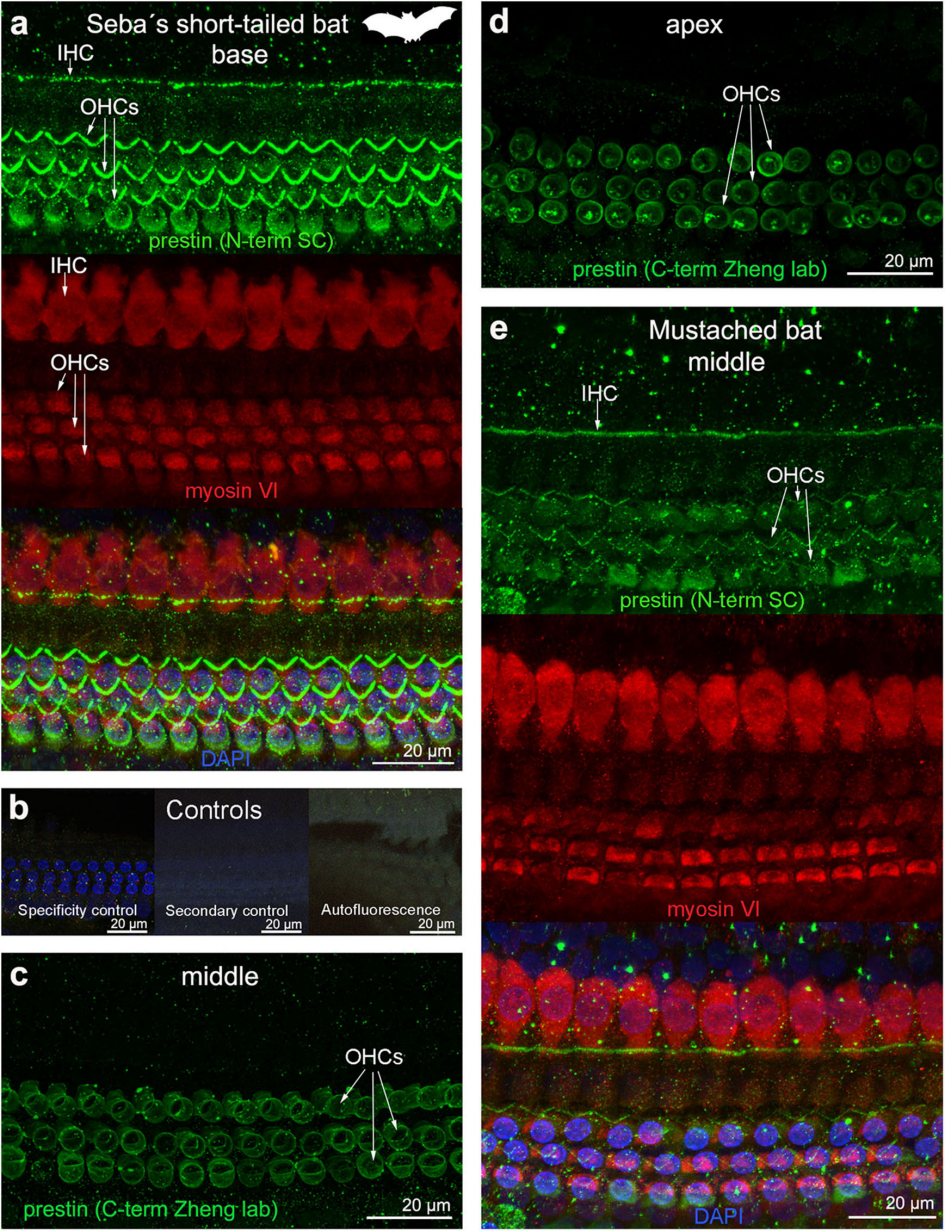
Immunofluorescence images from maximum projections from z stacks from several locations of the cochlea of **(a–d)** Seba's short-tailed bat (*Carollia perspicillata*) and **(e)** Mustached bat (*Pteronotus parnellii*). We labeled the whole mounts with anti-prestin antibody from Santa Cruz (SC) in **(a,e)** (green, 1:100 for Seba's short tailed bat and 1:200 for Mustached bat), with anti-prestin antibody from Dr. Zheng lab (green) in **(c,d)**, anti-myosinVI antibody (red) that labels inner (IHCs) and outer hair cells (OHCs), and DAPI (blue). **(b)** Specificity control, secondary control (control for non-specific binding of the secondary antibodies) and autofluorescence control from **(a)**. The cuticular plate from IHCs and OHCs was positively labeled by anti-prestin antibody from Santa Cruz, but not with anti-prestin antibody from Dr. Zheng lab. The animal image inserted in **(a)** corresponds to the animal species of cochlea stained by immunofluorescence.

The 19 cochleas from the five toothed whale species (harbor porpoise, bottlenose dolphin, common dolphin, striped dolphin, and beluga whale) showed some post-mortem decomposition artifacts, probably due to the delay between death and fixation of the cochlea (Table 1). In some cases, the whole cochlear turn was not intact and there were some areas where there were no remains of the cells of the organ of Corti, or the organ of Corti was not complete (i.e., the full three rows of OHCs and one row of IHCs were not in optimal condition state, and some cells were not present due to post-mortem decomposition). In spite of this, positive labeling for prestin was observed in OHCs basolateral membrane in toothed whales (Figures 2–4), as well as in the two species of bats (Parnell's mustached bat and Seba's short-tailed bat, Figure 5) all along the cochlear spiral. Although we only show here the results for harbor porpoise (Figure 2), beluga whale (Figure 3), bottlenose dolphin (Figure 4) and Seba's short tailed and mustached bat (Figure 5), we obtained the same pattern in the other species. However, since the tissue was less well-preserved in the other species, there were regions without labeling of the organ of Corti, or at least not in all the sensory cell rows. In spite of some degree of post-mortem decomposition, the specimens were generally of high quality for this investigation.

The prestin and myosin VI labeling showed that the organ of Corti was formed by one row of IHC and three rows of OHCs in echolocating whales and bats (Figures 2–5), as is typical in mammals and described previously by electron microscopy (30, 31, 42) and histology (43, 44). Thus, although decomposition artifacts were present, the tissue was still in good enough condition for immunofluorescence staining.

OHC stereocilia and cuticular plates, as well as IHC stereocilia (whenever present) and cuticular plates, identified with the myosin VI antibody, also stained positive for prestin in both species of bats and the five species of echolocating whales when using the antibody specific from the n-terminus extreme of the protein prestin from the company Santa Cruz. This unexpected labeling was not observed when using the 2 antibodies courtesy of Dr. Zheng's lab (recognizing the n- and c-terminus extremes, respectively, Figures 2d,e), indicating that the initial positive labeling for prestin in stereocilia and cuticular plates was due to non-specific binding of the antibody from Santa Cruz.

All controls (specificity control, secondary control, and blank) were negative (Figures 2–5).

### Combining Anti-prestin, Anti-myosin VI, Anti-neurofilament Antibody, and/or Phalloidin Can Distinguish Between Newly Formed Lesions and Old Ones

#### Anti-myosin VI Antibody

**Figure 6:**
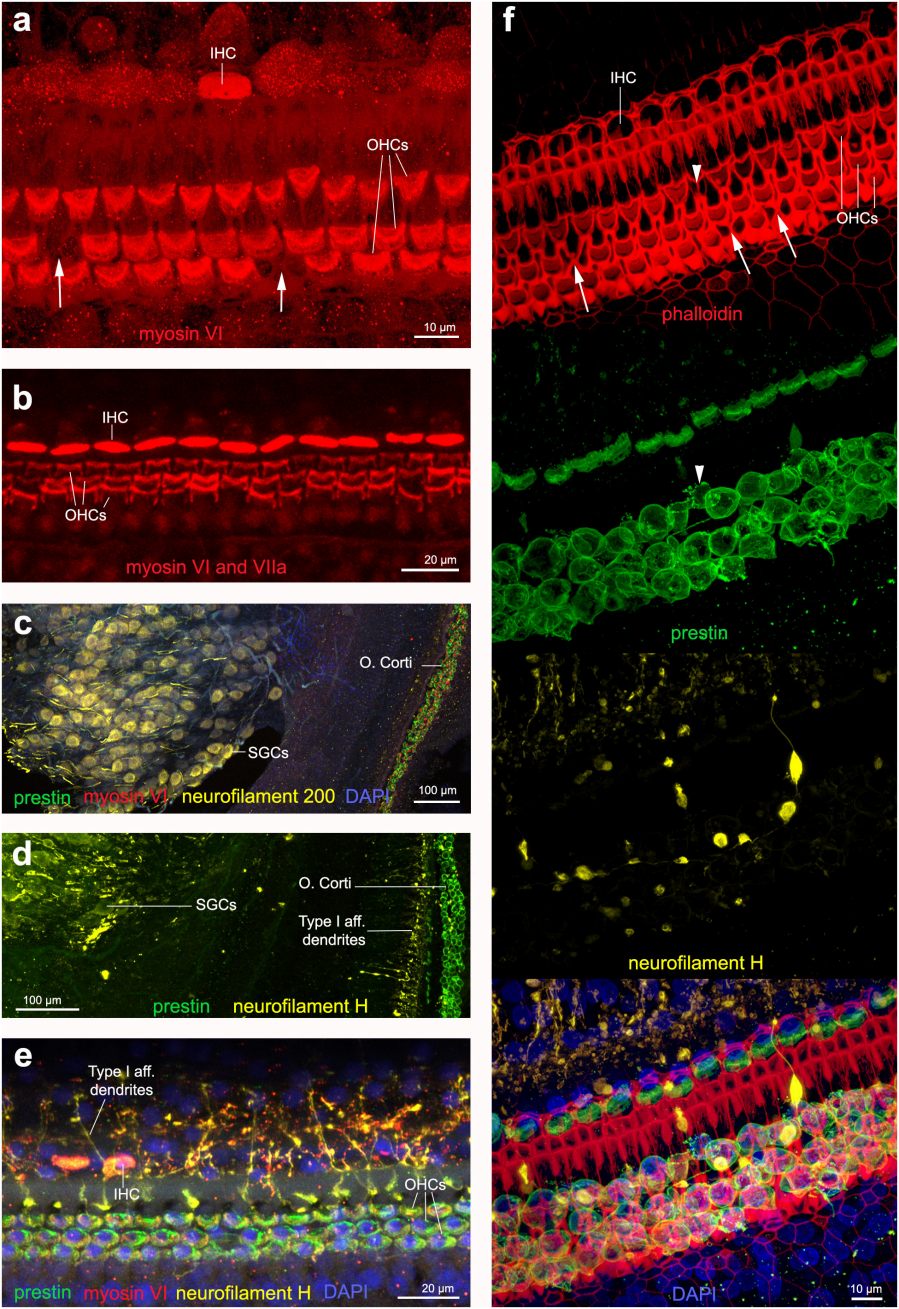
Immunofluorescence images from maximum projections from z stacks from harbor porpoise **(a–d,f)** and bottlenose dolphin **(e)** cochlea. **(a)** Labeling of the sensory cells with anti-myosin VI antibody. The arrows in **(a)** show the location of a scar. **(b)** Labeling of the sensory cells with anti-myosin VI and anti-myosin VIIa antibody, increasing the signal. Type I afferent innervation labeled by anti-neurofilament 200 **(c)** and anti-neurofilament H **(d)** antibodies. Note that while anti-neurofilament 200 antibody labels the spiral ganglion cells, anti-neurofilament H antibody also labels the dendrites in contact with inner hair cells. **(e)** Organ of Corti labeled with our recommended combination of antibodies: anti-prestin, myosin VI, neurofilament H and DAPI. **(f)** Organ of Corti labeled with phalloidin, anti-prestin, anti-neurofilament H, and all together in addition with DAPI, as we recommend for those samples fixed with 4% paraformaldehyde. The arrows highlight those older scars with shape of butterfly or sand clock and the arrow head points a scar in formation, with still part of the outer hair cell body as labeled by anti-prestin antibody.

Anti-myosin VI antibody proved to positively label the sensory cells. Stronger labeling was observed in the cuticular plate than the cytoplasm with our dilution (1:500). Whenever scars were present, it was possible to visualize them (arrows in Figure 6a). We observed weak (nearly perceptible) labeling with anti-myosin VIIa antibody (1:500 dilution), which is commonly used to label sensory cells in rodents. We also tried double labeling with myosin VI and myosin VIIa antibodies, leading to a much stronger signal, especially in the cuticular plates of IHCs and OHCs (Figure 6b).

#### Phalloidin

When the cochleas were fixed with 4% paraformaldehyde, phalloidin marked the reticular lamina of the sensory and supporting cells of the organ of Corti (Figure 6f). When the cochleas were fixed with 10% neutral buffered formalin, there was no positive labeling with phalloidin. Phalloidin labeling proved to be a good marker to distinguish among first stages of OHC death (arrowhead in Figure 6f) that had a different shape than older scars (arrows in Figure 6f).

#### Anti-neurofilament Heavy Chain Antibody

We tested two antibodies to label afferent type I innervation (i.e., nerves that transmit the auditory information from the IHCs to the brain): (1) mouse anti-neurofilament 200 kD from Sigma and (2) chicken anti-neurofilament H from Millipore. Both labeled positively the spiral ganglion neurons (i.e., the cell body of the type I neurons) and the auditory nerve. However, the neurofilament antibody raised in chicken also labeled the dendrites at the base of the IHCs in cetacean and bat species (Figures 6c,d). The neurofilament antibody raised in mouse also labeled positively the dendrites in the species of echolocating bats (not shown here), but not in cetaceans tested in our study.

In all the samples analyzed for this study, there were no apparent differences in staining patterns between juveniles or adults, nor evidences of noise-induced hearing loss. The combination of antibodies presented here also allowed describing the changes in morphology of the sensory cells of the organ of Corti along the cochlear spiral, which is better represented for harbor porpoise (Figure 2) and beluga whale (Figure 3) in our study.

## Discussion

In all the odontocete and bat cochleas used in this study, positive labeling for prestin was identified in the basolateral membrane of OHCs throughout the cochlear spiral, including in the basal turn. This finding strongly suggests that echolocating whales and bats express the protein prestin, as do other mammals (2, 3).

The presence of prestin in echolocating species is consistent with the predictions by Johnson et al. (45) that the protein may function as a cochlear amplifier for high frequency sounds. A major limitation in membrane potential changes on a cycle-by-cycle basis is the OHC membrane time constant, which is responsible for low-pass filtering. However, at physiological endolymphatic calcium concentrations, there is little receptor potential attenuation at the characteristic frequency of the OHC, which suggests a minimal time constant filtering *in vivo* and an optimal activation of prestin over the entire range of hearing in mice (45). It is thus reasonable that echolocating species are using prestin-driven electromotility to encode high-frequency stimulation. In addition, the extremely short length of the OHCs in the most basal portion of the cochlea in echolocating species, reaching 8 μm for harbor porpoises (30) and bicolored round-leaf bats [*Hipposideros bicolor*, (46)], would contribute to a shorter membrane time constant and extend the frequency range in which prestin would operate. Because of the positive labeling of prestin in the most basal turn of the cochlea, our results provide morphological evidence that prestin can have a role in cochlear amplification in the basolateral membrane up to 120–180 kHz in our odontocete and bat subjects, which is the highest frequency reported at this time.

The unusual positive labeling of prestin in the cuticular plate and stereocilia of inner and outer hair cells found in goat anti-prestin antibody from Santa Cruz (SC-22692) proved to be due to non-specific binding of this antibody, since the two antibodies against prestin raised in rabbit provided by Dr. Zheng lab (NW802 and NW958) did not show this labeling pattern. Both antibodies SC-22692 and NW802 are polyclonal and recognize the 50 and 20 first amino acids of the N-terminus extreme of the protein prestin in humans and mice, respectively, while NW958 recognizes the last 20 amino acids of the c-terminus extreme of the protein in mice. The gene for prestin has been fully sequenced for harbor porpoise (GenBank GU219842.1), for bottlenose dolphin (GenBank GU217587.1), common dolphin (GenBank GU219839.1) and partially sequenced for mustached bat (GenBank JN315991.1; the n-terminus extreme is not complete) (14, 47). The full sequence has also been predicted by automated computational analysis using gene predicting method Gnomon, supported by mRNA evidence for beluga whale (NCBI reference sequence XM_022554419.1). The prestin gene shows 86% homology with the N-terminus extreme between harbor porpoise, beluga whale, bottlenose, and common dolphins and humans (50 first amino acids) and 80% homology with mice (20 first amino acids). It also has 85 and 75% homology with the c-terminus extreme with a deletion of three amino acids (in the 20 last amino acids) between these echolocating whales and mustached bat, respectively, and mice. The reason of the differential results among antibodies is unclear, but since they have never been tested in these species of cetaceans or bats, it is possible to discover that some antibodies recognize other structures, apart from prestin. The specificity of the antibody from Santa Cruz was determined in a peptide neutralization assay with peptide sc-22692 P that corresponds to amino acids 1–50 of human prestin. The specificity of the antibodies from Dr. Zheng lab to recognize the protein prestin were tested with ELISA, western blot and immunofluorescence experiments performed on prestin-expressing samples, as well as with the use of prestin-knockout mice (39, 40). We have combined the results of several antibodies, raised in different species and that recognize different sequences of the protein prestin to discriminate between non-specific binding.

Prestin labeling can be used in cetaceans to detect potential cases of acute noise-induced hearing loss, specifically if the individuals have died in the 9 days following the exposure, as has previously been shown in rodents (25), assuming that the scarring process takes place at the same rate in cetaceans. Phalloidin labeling is optimal to detect cases of OHC loss within the first 9 h post-exposure (32). As shown in Figure 6f, the arrows highlight scars that correspond to same shape (like a butterfly or hour-glass) than those over 9 h post-exposure in guinea pigs. However, the scar shown with the arrowhead would represent a more recent case, with a strong similarity to the shape that scars have around 6 h post-exposure in guinea pigs, with a central thicker vertical line and two fainter nearly parallel lines on the side (32). With the prestin labeling it is possible to observe that the outer hair cell body is still deeper in the tissue, but not reaching the cuticular lamina, as an indication to a scar that is still in the formation process. Further labeling with markers for apoptosis and autophagy should be done to verify whether it is a case of hair cell death by apoptosis. In any case, we demonstrated here that phalloidin labeling is optimal for detecting recent cases of hearing loss. However, the inner ears need to be fixed with 4% paraformaldehyde, which is not a common fixative among cetacean stranding networks because of its difficulty of preparation and storage. There was no positive labeling of phalloidin when the samples were fixed with 10% neutral buffered formalin likely because formalin contains numerous compounds other than formaldehyde that likely either destabilize actin or interfere with phalloidin binding [for example, methanol that is often present in formalin, denatures F-actin (48)]. We also were unsuccessful using Sudan Black B to decrease the autofluorescence of the tissue, possibly because it contains 70% ethanol, which is known to depolymerize F-actin (49, 50).

The personnel responsible of performing necropsies on cetaceans use 10% neutral buffered formalin to fix all tissues for histopathological analysis (51). Thus, phalloidin labeling is not always an option when collecting and fixing the cochlea. Instead of using phalloidin, anti-myosin VI antibody is a suitable candidate to mark the hair cells. The labeling of the cuticular plate and stereocilia of hair cells was stronger than in the cell body with anti-myosin VI antibody. The reason of this difference in labeling is not clear, but may be explained by the fact that the cuticular plate is more resistant to post-mortem decomposition. Thus, the faint labeling of the hair cell body could be an indication of the first stages of cell decomposition.

In the cetacean species of this study, while the neurofilament 200 antibody (raised in mouse) labeled the spiral ganglion cells (Figure 6c), the neurofilament H antibody (raised in chicken) also labeled the dendrites in contact with inner hair cells (Figures 6d–f). Based on these results, we recommend using the anti-neurofilament H antibody for the analysis of the cochleas of stranded cetaceans as it may allow detecting potential cases of degeneration of the dendrites of type I afferent neurons as a consequence of noise exposure. The presence of spiral ganglion neurons in Figure 6f could be an artifact of the dissection and mounting of the whole-mount preparation, where a few neurons detached and moved below the cells of the organ of Corti.

There is an urgent need to develop methods for assessing the effects of underwater anthropogenic noise on cetaceans. The use of antibodies for immunofluorescence depends on the target species and little research has been done on cetacean tissue. Here, we report a method optimized for cetacean cochlea that allows the visualization of the hair cells and supporting cells of the organ of Corti, as well as type I innervation by combining several antibodies. The combination of anti-prestin, anti-neurofilament H, anti-myosin VI antibodies and/or phalloidin also distinguishes between newly formed and old lesions.

## Data Availability Statement

The original contributions presented in the study are included in the article/supplementary material, further inquiries can be directed to the corresponding author/s.

## Ethics Statement

The permits required for the experiments with cetaceans were obtained as follows: Aurora Research Institute License No. 15467, Department of Fisheries and Oceans Fishing License No. S-14/15-3019-YK, Marine Mammal Transport License #18843, and Environmental Impact Screening Committee (EISC) #03-14-03. All protected cetacean samples were transported with the appropriate CITES permits. All experiments with bats were performed in accordance with current laws for animal experimentation in Germany (Regierungspräsidium Darmstadt) and with the Declaration of Helsinki.

## Author Contributions

MM, LI, MP-D, SO, MF, NF-F, KC, and SR were in charge of the necropsies from cetaceans and to remove and fix the ears. MM performed the inner ear dissection, their preparation for confocal microscopy, and image interpretation. LT and AV supervised the dissection and processing protocols for whole-mount preparations. MM, AV, LI, MP-D, LT, SO, MF, NF-F, KC, J-LP, SR, and RS helped with writing and editing the manuscript. RS, SR, AV, and J-LP supervised the work and its publication.

## Conflict of Interest

The authors declare that the research was conducted in the absence of any commercial or financial relationships that could be construed as a potential conflict of interest.
